# Supplementary material for: From sequence to enzyme mechanism using multi-label machine learning
Source: BMC Bioinformatics. 2014 May 19;15:150. doi: 10.1186/1471-2105-15-150 (PMC4229970; doi:10.1186/1471-2105-15-150)
Supplement: Additional file 2 — Java code of ml2db. Additional file ml2db_code.tar.gz contains the Java source code to run the multi-label machine learning experiments and save the results to database. The code’s Javadoc is included. [file 1471-2105-15-150-S2.zip › additional file 2/ml2db/ml2db/doc/mulan/data/LabelNodeImpl.html]

LabelNodeImpl


JavaScript is disabled on your browser.


- Overview
- Package
- Class
- Use
- Tree
- Deprecated
- Index
- Help

- Prev Class
- Next Class

- Frames
- No Frames

- All Classes

- Summary:
- Nested |
- Field |
- Constr |
- Method

- Detail:
- Field |
- Constr |
- Method


mulan.data

## Class LabelNodeImpl

- java.lang.Object
- - mulan.data.LabelNodeImpl

- All Implemented Interfaces:
  :   java.io.Serializable, LabelNode

  ---

    

  ```
  public class LabelNodeImpl
  extends java.lang.Object
  implements LabelNode, java.io.Serializable
  ```

  Implementation of `LabelNode`, representing a label attribute and its connection
  within a hierarchy of labels.

  Author:
  :   Jozef Vilcek

  See Also:
  :   Serialized Form

- - ### Constructor Summary

    Constructors

    Constructor and Description

    `LabelNodeImpl(java.lang.String name)`

    Creates a new instance of `LabelNodeImpl`.
  - ### Method Summary

    Methods

    | Modifier and Type | Method and Description |
    | --- | --- |
    | `boolean` | `addChildNode(LabelNode node)` Adds the specified `LabelNode` to the set of child nodes. |
    | `boolean` | `equals(java.lang.Object obj)` The two `LabelNodeImpl` nodes are equal if the are the same (points to the same object) of if they returns same `getName()` value. |
    | `java.util.Set<LabelNode>` | `getChildren()` Gets the unmodifiable `Set` of child `LabelNode` of this node, if hierarchy exists. |
    | `java.util.Set<java.lang.String>` | `getChildrenLabels()` Gets the children of a label |
    | `java.util.Set<java.lang.String>` | `getDescendantLabels()` Gets a `Set` of the names of descendant `LabelNode` of this node, if hierarchy exists. |
    | `java.lang.String` | `getName()` Gets the name of the label this node represents. |
    | `LabelNode` | `getParent()` Gets the parent `LabelNode` of this node if hierarchy exists. |
    | `boolean` | `hasChildren()` Determines whether the `LabelNode` has child nodes. |
    | `int` | `hashCode()` The hash code is computed based on label name attribute, which defines the identity of the `LabelNodeImpl` node. |
    | `boolean` | `hasParent()` Determines whether the `LabelNode` has a parent node in a hierarchy. |
    | `boolean` | `removeChildNode(LabelNode node)` Removes the specified `LabelNode` from the set of child nodes. |

    - ### Methods inherited from class java.lang.Object

      `getClass, notify, notifyAll, toString, wait, wait, wait`

- - ### Constructor Detail


    - #### LabelNodeImpl

      ```
      public LabelNodeImpl(java.lang.String name)
      ```

      Creates a new instance of `LabelNodeImpl`.

      Parameters:
      :   `name` - the name of the label attribute this node represents
  - ### Method Detail


    - #### addChildNode

      ```
      public boolean addChildNode(LabelNode node)
      ```

      Adds the specified `LabelNode` to the set of child nodes.
      The parent of added node is set to reference this `LabelNode` instance.
      This indicates that there is a hierarchy between these two `LabelNode` nodes.

      Parameters:
      :   `node` - the `LabelNode` to be removed

      Returns:
      :   true if node was actually removed; false node was not in child nodes set

      Throws:
      :   `ArgumentNullException` - if specified `LabelNode` parameter is null
      :   `java.lang.IllegalArgumentException` - if `LabelNode` being added has same name as this `LabelNode` instance (parent)


    - #### removeChildNode

      ```
      public boolean removeChildNode(LabelNode node)
      ```

      Removes the specified `LabelNode` from the set of child nodes.
      The connection between removed `LabelNode` and its `LabelNode.getParent()`

      Parameters:
      :   `node` - the `LabelNode` to be removed

      Returns:
      :   true if node was actually removed; false node was not in child nodes set

      Throws:
      :   `ArgumentNullException` - if specified `LabelNode` parameter is null


    - #### getChildrenLabels

      ```
      public java.util.Set<java.lang.String> getChildrenLabels()
      ```

      Gets the children of a label

      Returns:
      :   a Set of labels


    - #### getDescendantLabels

      ```
      public java.util.Set<java.lang.String> getDescendantLabels()
      ```

      **Description copied from interface: `LabelNode`**

      Gets a `Set` of the names of descendant `LabelNode` of this node, if hierarchy exists.
      If no descendant nodes exists for this `LabelNode`, empty `Set` is returned.

      **Specified by:**
      :   `getDescendantLabels` in interface `LabelNode`

      Returns:
      :   the `Set` of child nodes


    - #### getChildren

      ```
      public java.util.Set<LabelNode> getChildren()
      ```

      **Description copied from interface: `LabelNode`**

      Gets the unmodifiable `Set` of child `LabelNode` of this node, if hierarchy exists.
      If no child nodes exists for this `LabelNode`, empty `Set` is returned.

      **Specified by:**
      :   `getChildren` in interface `LabelNode`

      Returns:
      :   the `Set` of child nodes


    - #### getName

      ```
      public java.lang.String getName()
      ```

      **Description copied from interface: `LabelNode`**

      Gets the name of the label this node represents.
      The name corresponds to the ID of label attribute in the arff data set.
      The name of the label must be unique within the data set, because the
      identity of `LabelNode` is determined by the name.

      **Specified by:**
      :   `getName` in interface `LabelNode`

      Returns:
      :   the name of the label this node represents


    - #### getParent

      ```
      public LabelNode getParent()
      ```

      **Description copied from interface: `LabelNode`**

      Gets the parent `LabelNode` of this node if hierarchy exists.
      If the node has not a parent `LabelNode`, `null` is returned.

      **Specified by:**
      :   `getParent` in interface `LabelNode`

      Returns:
      :   the parent `LabelNode` or `null` if the parent does not exists.


    - #### hasChildren

      ```
      public boolean hasChildren()
      ```

      **Description copied from interface: `LabelNode`**

      Determines whether the `LabelNode` has child nodes.

      **Specified by:**
      :   `hasChildren` in interface `LabelNode`

      Returns:
      :   `true` if the node has child nodes; `false` otherwise.


    - #### hasParent

      ```
      public boolean hasParent()
      ```

      **Description copied from interface: `LabelNode`**

      Determines whether the `LabelNode` has a parent node in a hierarchy.

      **Specified by:**
      :   `hasParent` in interface `LabelNode`

      Returns:
      :   `true` if the node has parent; `false` otherwise.


    - #### hashCode

      ```
      public int hashCode()
      ```

      The hash code is computed based on label name attribute, which defines the
      identity of the `LabelNodeImpl` node.

      **Overrides:**
      :   `hashCode` in class `java.lang.Object`


    - #### equals

      ```
      public boolean equals(java.lang.Object obj)
      ```

      The two `LabelNodeImpl` nodes are equal if the are the same
      (points to the same object) of if they returns same `getName()` value.
      The name of the labels gives the identity to the `LabelNodeImpl`.

      **Overrides:**
      :   `equals` in class `java.lang.Object`


- Overview
- Package
- Class
- Use
- Tree
- Deprecated
- Index
- Help

- Prev Class
- Next Class

- Frames
- No Frames

- All Classes

- Summary:
- Nested |
- Field |
- Constr |
- Method

- Detail:
- Field |
- Constr |
- Method
